# Contribution of n-3 Long-Chain Polyunsaturated Fatty Acids to the Prevention of Breast Cancer Risk Factors

**DOI:** 10.3390/ijerph19137936

**Published:** 2022-06-28

**Authors:** Mostefa Fodil, Vincent Blanckaert, Lionel Ulmann, Virginie Mimouni, Benoît Chénais

**Affiliations:** 1 Biology of Organisms: Stress, Health, Environment (BiOSSE), UFR Sciences et Techniques, Le Mans Université, F-72085 Le Mans, France; mostefa.fodil@univ-lemans.fr; 2 Biology of Organisms: Stress, Health, Environment (BiOSSE), Institut Universitaire de Technologie, Département Génie Biologique, Le Mans Université, F-53020 Laval, France; vincent.blanckaert@univ-lemans.fr (V.B.); lionel.ulmann@univ-lemans.fr (L.U.); virginie.mimouni@univ-lemans.fr (V.M.)

**Keywords:** docosahexaenoic acid, eicosapentaenoic acid, cancer therapy, cancer prevention, angiogenesis, apoptosis, cell migration, cell proliferation

## Abstract

Nowadays, diet and breast cancer are studied at different levels, particularly in tumor prevention and progression. Thus, the molecular mechanisms leading to better knowledge are deciphered with a higher precision. Among the molecules implicated in a preventive and anti-progressive way, n-3 long chain polyunsaturated fatty acids (n-3 LC-PUFAs) are good candidates. These molecules, like docosahexaenoic (DHA) and eicosapentaenoic (EPA) acids, are generally found in marine material, such as fat fishes or microalgae. EPA and DHA act as anti-proliferative, anti-invasive, and anti-angiogenic molecules in breast cancer cell lines, as well as in in vivo studies. A better characterization of the cellular and molecular pathways involving the action of these fatty acids is essential to have a realistic image of the therapeutic avenues envisaged behind their use. This need is reinforced by the increase in the number of clinical trials involving more and more n-3 LC-PUFAs, and this, in various pathologies ranging from obesity to a multitude of cancers. The objective of this review is, therefore, to highlight the new elements showing the preventive and beneficial effects of n-3 LC-PUFAs against the development and progression of breast cancer.

## 1. Introduction

High saturated fat consumption is commonly associated with an increased risk for several types of diet-related cancers, such as breast [1,2], colonic [3], or pancreatic tumors [4]. Breast cancer can be classified in different subtypes based on histological information, Nottingham grade, hormone receptor status, and human epidermal growth factor receptor 2 (HER2) status [5]. Transcriptomic analysis further evidenced additional heterogeneity among breast cancers, and a total of five major subgroups are identified based on RNA expression profiles, namely, luminal A, luminal B, HER2-enriched, low claudin, and basal-like [6,7]. Estrogen receptor alpha (ERα)-positive breast cancers are mainly related to luminal A and B, whereas HER2-positive and triple-negative breast cancers (TNBC) are mainly related to HER2-enriched and basal-like, respectively. In general, the aggressiveness of luminal A cancers is lower than that of luminal B cancers, as they show lower expression of Ki67, which is a marker of proliferation, and expression of HER2 in addition to ERα [6,7]. TNBCs are lacking the three biomarkers, i.e., HER2, Erα, and progesterone receptors, and do not respond to hormone therapy for instance. Not all TNBCs are basal-like subtypes, and they can be divided into six subtypes: (i) basal-like-1 (BL1), (ii) BL2, (iii) immunomodulatory subtype, (iv) mesenchymal subtype, (v) mesenchymal stem-like subtype, and (vi) luminal androgen receptor subtype, each of which has distinct gene expressions and ontologies [8]. Therefore, the existence of different subtypes might suggest a differential response to a given treatment, such as chemotherapy, radiotherapy, hormone therapy, or surgery. However, the occurrence of drug resistance and side effects decrease their efficiencies [9]. For this reason, it is crucial to find natural molecules acting as anti-cancer and/or anti-invasive compounds that particularly lessen the side effects of breast cancer. Besides, breast cancer is the most frequent cancer and the major cause of death in women in industrialized countries. Diet and nutrition are environmental factors that are likely to have an influence on health [10], and particularly on preventive breast tumors’ emergence [11]. Furthermore, epidemiological studies highlighted a 4-to-5-fold greater rate of breast cancer in Western countries than in Japan, associated, at least in part, with a diet low in fatty fish rich in polyunsaturated fatty acids (PUFAs) [12]. These observations suggest that diets rich in n-3 and n-6 long-chain PUFAs (LC-PUFAs) may help reduce cancer risk [13,14]. However, though dietary n-3 LC-PUFAs appear to have a preventive effect on cancer, n-6 LC-PUFAs have been associated with a higher risk of induction of tumor formation and inflammatory processes [1,13,15]. This shows the importance of the n-3:n-6 ratio in breast cancer prevention [13].

Hence, in this review, we will focus on n-3 LC-PUFAs at different biological and molecular levels using in vitro and in vivo breast cancer models, including antiproliferative and pro-apoptotic effects, anti-invasive and anti-metastatic effects, modulation of angiogenesis, and anti-inflammatory effect in the context of breast cancer. The preventive effect of n-3 LC-PUFAs has also been demonstrated in clinical studies, and their potential as an adjuvant to conventional therapy is also highlighted here. 

## 2. Antiproliferative and Pro-Apoptotic Effects of n-3 LC-PUFAs

The antiproliferative and pro-apoptotic effects of n-3 LC-PUFAs have been extensively studied in many human cancer cell lines and using purified n-3 LC-PUFAs, mainly DHA [16,17,18,19,20]. However, understanding the cellular and molecular mechanisms of the anticancer effect of n-3 LC-PUFAs remains an important area of current research, and despite numerous efforts, it remains difficult to identify a clear pattern of the involved mechanisms. Indeed, important differences exist between the different cell types or cell lines used, and also in the experimental conditions (concentration, exposure time, co-treatments, etc.).

**Figure 1:**
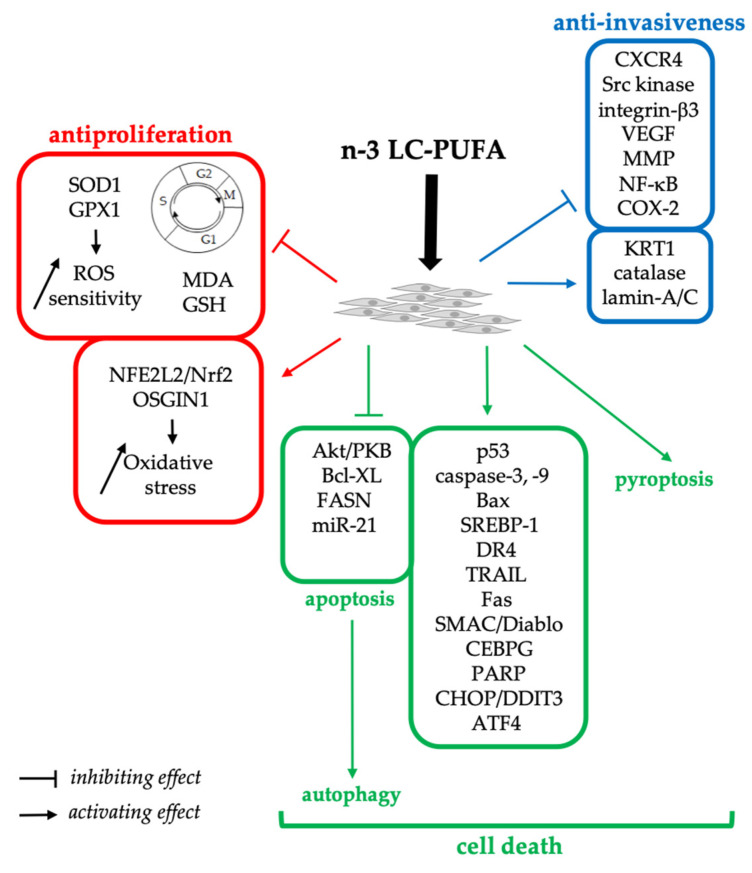
Antiproliferative (red), pro-apoptotic (green), and anti-invasive (blue) effects of n-3 LC-PUFA on human breast cancer cell lines. This figure summarizes different markers of target genes or proteins and signaling pathways involved in the regulation of mechanisms involved in the inhibition of breast cancer development. MDA, malondialdehyde; GSH, reduced glutathione; ROS, reactive oxygen species; other abbreviations are gene/protein names.

In various breast cancer cell lines, DHA acted as an anti-proliferative agent by lengthening the cell cycle, leading to cell cycle arrest at the G1 or G2/M phase (Figure 1) [21]. Increased sensitivity to reactive oxygen species (ROS) in DHA-treated cells, especially through downregulation of superoxide dismutase 1 (SOD1) and glutathione peroxidase 1 (GPX1), has also been invoked as an explanation for the antiproliferative effect of DHA [16,18,22]. In addition, DHA was also reported as inducing the expression and nuclear translocation of the oxidative stress sensitive transcription factor, NFE2L2/Nrf2, and to increase the expression of oxidative stress-induced growth inhibitor 1 (OSGIN1) in MCF-7 and Hs578T breast cancer cells (Figure 1) [23]. However, the growth of MCF-7 cells was reduced by using extract from the microalga Chlorella sp. S14, which contains about 12% of n-3 LC-PUFAs, including ~2% DHA and ~2% EPA, whereas this extract displays antioxidant capacities by reducing malondialdehyde and glutathione levels, and increasing catalase activity [24]. The effect of DHA in breast cancer tissues (by opposition to cell lines) also leads to an increase in antioxidant enzyme activity (SOD, GPX, and catalase) [25]. Altogether, these results suggest that the anti/pro-oxidant effects of DHA may depend on the cell type, concentration, and formulation used.

Most studies reported increased cell death due to apoptosis after n-3 LC-PUFA treatment of breast cancer cells, and few reported autophagy [26,27] or pyroptosis [28]. It is worth noting that n-3 LC-PUFAs do not appear to have pro-apoptotic effects on non-tumor cells, as has been shown with the human breast epithelial cell lines, MCF-10A and H184-A1N4 [23,26].

Apoptosis was induced by DHA, and in a lesser extent by EPA, in cancer cells through impairment of the activity of protein kinase, Akt/PKB; p53 activation; increased caspase-3, caspase-9, and Bax pro-apoptotic enzyme levels or activity; and decreased survival and Bcl-XL, with DHA or EPA concentration ranging from 10 to 200 µM (reviewed in: [16,21]). The inactivation of the PI3K/Akt pathway is the main apoptosis pathway mentioned in the literature (Figure 1) [16,21]. For example, Akt signaling was inhibited by DHA in MCF-7 cells, leading to decreased fatty acid synthase (FASN) expression and reduced cell proliferation [29]. In addition, DHA inhibits the expression of the transcription factor, SREBP-1, induced by either estradiol or insulin [29]. Still, in the MCF-7 cell line, DHA-induced apoptosis was linked to both increased death receptors (DR-4, TRAIL, and Fas) expression and mitochondrial release of the caspase activator, SMAC/Diablo [30]. DHA also induced a time-dependent decrease of the miR-21 oncogenic microRNA, but without a significant change in its molecular target PTEN expression [31]. Recently, 1-docosahexaenoyl glycerol, then called DHA-monoglyceride, was shown to induce cell growth inhibition and apoptosis by means of increased active forms of caspase-3, caspase-12, and poly-ADP-ribose polymerase (PARP) in both MCF-7 and MDA-MB-231 breast cancer cell lines [27]. In this study, DHA-monoglyceride-induced apoptosis was triggered by the endoplasmic reticulum (ER) stress pathway, and inhibited by the antioxidant, N-acetyl-cysteine [27]. The involvement of ER-stress was evidenced by increased amounts of phosphorylated PERK, phosphorylated e-IF2α, and CHOP/DDIT3 in both cell lines. The stimulation of the pro-apoptotic ER-stress pathway, and especially the overexpression of CHOP/DDIT3 and ATF4, was also reported in the transcriptomic analysis of DHA-treated MDA-MB-231 cells [32]. This study also showed the upregulation of several other pro-apoptotic genes, such as the transcription factor, CEBPG, which may stimulate the Akt-dependent apoptotic pathway; the G0S2 protein, which is known to bind BCL-2; and the GADD34 and DDIT4 protein, which are both linked to the p53-mediated apoptosis [32]. In addition, this study highlighted the inhibitory effect of DHA treatment on lipogenesis, especially through downregulation of FASN and cholesterol’s biosynthesis pathway [32].

Autophagy was also reported to be induced by DHA-monoglyceride and dopamine-conjugated DHA and EPA in MCF-7 and MDA-MB-231 cell lines [26,27]. However, though both studies showed the presence of both autophagy and apoptosis, apoptosis appears to precede (and be inhibited by) autophagy in cells treated with DHA-monoglyceride [27], whereas apoptosis appears after autophagy with dopamine conjugates [26]. Studies on autophagy are still too few, and more results, especially with unconjugated DHA, are needed to draw reliable conclusions.

Finally, one study reported that DHA induces pyroptosis in the MDA-MB-231 breast cancer cell line with a usual concentration of 100 µM DHA and exposure time of 18 h, a bit shorter than usual experiments [28]. Pyroptosis is a form of programmed cell death mediated by inflammatory caspases (i.e., caspase-1, -4, -5 in humans), and leads to pore formation in the cell membrane by the N-terminal region of Gasdermin D, all markers that have been observed in DHA-treated cells [28].

Although both cell lines are breast cancer cell lines, MDA-MB-231 is “basal” and triple negative (ER-, PR-, and HER2-negative), and MCF7 is “luminal” and ER- and PR-positive. Thus, different breast cancer subtypes may have different molecular environments, including oxidative stress sensitivity and ROS-dependent regulation of the stress response transcription factor, NFE2L2, which is related to estrogen response [33,34]. Such differences could affect drug sensitivity and therapeutic efficacy, and should be considered.

## 3. Protective Effect of Nutritional n-3 LC-PUFAs against Breast Cancer in Animal Models

**Figure 2:**
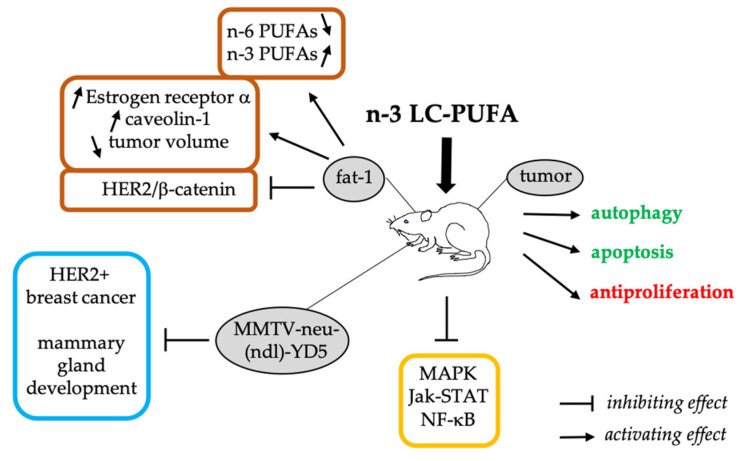
Protective effects of dietary n-3 LC-PUFAs in animal models of breast cancer.

Several studies have been conducted in animal models of chemically-induced tumors or tumor xenografts that reported antiproliferative and pro-apoptotic effects of DHA or dietary fats (reviewed in: [16,35,36,37,38]). In addition, Wang et al. recently reported the induction of autophagy in breast tumor cells from xenografted mice administered intragastrically with DHA-monoglyceride daily [27]. Moreover, the potential preventive effects of n-3 LC-PUFAs against numerous types of cancer have been reported using the fat-1 transgenic mouse that expresses n-3 desaturase, missing in mammalian cells. Thus, fat-1 transgenic mice are able to produce n-3 fatty acids from the n-6 ones, leading to abundant n-3 fatty acids with reduced levels of n-6 fatty acids in their organs and tissues, without the need of a dietary n-3 supply [39,40]. These studies mainly focused on colorectal cancer and colitis-associated cancer [38], but have also targeted other types of cancers, including breast cancer [16,40,41]. For example, Zou et al. showed the protective effect of n-3 LC-PUFAs (especially 15-hydroxy eicosapentaenoic acid, 17-hydroxy docosahexaenoic acid, and prostaglandin E3) against breast cancer in fat-1 mice versus wild-type mice [42]. This preventive effect was partly related to the repression of the HER2/β-catenin signaling pathway and production of significant levels of n-3 LC-PUFA-derived bioactive mediators in the tumors of fat-1 mice compared with wild-type mice [42] (Figure 2).

The influence of lifelong exposure to various dietary fats on mammary tumor development was investigated by using the mouse mammary tumor virus MMTV-neu(ndl)-YD5 model, which is a murine equivalent to human HER2+ breast cancer, over a 20-week period [43]. The results highlighted the influence of diet on mammary tumor outcome, and specifically showed that the occurrence of HER2+ breast cancer is attenuated by the consumption of n-3 polyunsaturated, saturated, and monounsaturated fatty acids compared to n-6 polyunsaturated fatty acids [43] (Figure 2). Previously, the same group had shown the beneficial effect of n-3 LC-PUFAs in reducing the risk of breast cancer by altering the early development of the mammary gland. Indeed, they showed that fat-1 mice had increased expression of ERα and caveolin-1 (2-fold and 1.5-fold, respectively) compared to wild-type mice [44]. Similarly, a comparison between the offspring of wild-type mice and fat-1 mice fed an n-6 PUFA diet during pregnancy showed that wild-genotype offspring of fat-1 mothers had lower tumor incidence and smaller tumor volume compared with wild-genotype offspring of wild-type mothers [45]. Moreover, this study showed that n-3 LC-PUFAs in mothers altered the expression of several long noncoding RNA (lncRNA) and signaling pathways (i.e., MAPK, Jak-STAT, and NF-κB) in the mammary gland of offspring [45]. These results, and similar ones obtained with wild-type C57BL/6J mice or transgenic C3(1) Tag mice, highlighted the influence of the mother’s diet on the occurrence and development of breast cancer in her offspring [45,46,47,48]. The molecular mechanisms of this protective effect of n-3 LC-PUFAs are still poorly understood, but a recent study has shown the involvement of gut microbiota restructuring as a mechanism of maternal n-3 LC-PUFAs’ protective effect against mammary tumors in offspring [47].

## 4. Anti-Invasive and Anti-Metastatic Effects of n-3 LC-PUFAs

Few studies have shown that the diet can affect the metastatic potential of breast cancer cells known to have a high metastatic phenotype [49,50]. In the breast cancer cell line, MDA-MB-231, DHA reduced the migration and invasive potential of cells at a concentration ranging from 10 to 100 µM [51,52,53,54]. The proteomic analysis of DHA-treated MDA-MB-231 cells evidenced three upregulated membrane proteins, namely, KRT1 (UniProt ID P04264), catalase (UniProt ID P04040), and lamin-A/C (UniProt ID P02545), as well as two unidentified ones [55] (Figure 1). The involvement of KRT1 (also known as cytokeratin-1) in DHA-mediated inhibition of cell invasion was further demonstrated by using siRNA, and it could involve its interaction with integrins such as integrin-α1 [55]. In addition, DHA may alter the biophysical properties of lipid rafts by decreasing the content of cholesterol and the distribution of key proteins, such as EGFR, Src, heterotrimeric G-protein subunits, or sphingomyelinase. Indeed, the disruption of lipid raft domains following DHA-treatment was shown in MDA-MB-231 cells, and was associated with a decreased surface expression of the chemokine receptor, CXCR4, and reduced cell migration [54]. Among the signaling proteins that may be affected, Src kinase may play an important role in regulating migration and invasion of the MDA-MB-231 cell line [56] (Figure 1). Src has been shown to play a role in cancer and invasiveness [56,57], and has also been linked to other molecules, such as KRT1, via integrin β1, or voltage-dependent Na+ channels, one of whose subunits can also be considered an integrin, and whose involvement in the beneficial effects of n-3 LC-PUFAs has been reported [58]. A reduced expression of integrin-β3, vascular endothelial growth factor (VEGF), and matrix metalloproteinase (MMP) -1 was reported in DHA-treated MDA-MB-231 cells (Figure 1), which is related to the inhibition of invasiveness and cell migration [52]. It can be noted that EPA, as well as endocanabinoid derivatives of DHA and EPA, also inhibit integrin-β3 expression and cell invasion, but not migration of MDA-MB-231 cells nor MMP1 and VEGF expression [52]. Furthermore, the decrease of MMP-2 and MMP-9 expression and activity in DHA-treated MDA-MB-231 cells was demonstrated, and this effect of DHA was shown to be related to the inhibition of the NF-κB pathway through IκB kinase inhibition, thereby decreasing NF-κB-mediated stimulation of the MMP promoter [53] (Figure 1). These authors also showed that DHA suppresses the expression and promoter activity of the MMP-2 and MMP-9 genes by inhibiting cyclooxygenase 2 (COX2) and prostaglandin-E2 (PGE2) production by competing with arachidonic acid (ARA) [53]. The downregulation of COX2 and decrease in the level of phosphorylated Src kinase were also observed in tamoxifen-resistant MCF-7 (TamR) cells treated with 0.1% (v:v) fish oil, along with MAPK and PI3K inhibitors (i.e., PD98059 and LY294002, respectively), whereas COX2 and phosphorylated Src were unchanged in cells treated with the inhibitors, but in the absence of fish oil [59]. This treatment of TamR cells with both fish oil and kinase inhibitors is associated with an inhibition of cell migration [59].

The mechanisms and signaling pathways involved in the anti-metastatic effects of DHA remain to be understood. However, this anti-metastatic effect is corroborated by in vivo animal studies using fat-1 transgenic mice that demonstrated a decrease of tumor growth and reduction of lung metastasis of syngeneic breast cancer cells (i.e., EO771 cells) in this DHA-rich environment [53].

## 5. Modulation of Angiogenesis by n-3 LC-PUFAs

Angiogenesis, the formation of new blood vessels, is an essential feature of malignant tumor development. It is well established now that n-3 LC-PUFA (in particular, EPA and DHA, found principally in oily fish) have potent anti-angiogenic effects inhibiting the production of many important angiogenic mediators, namely, VEGF, platelet-derived growth factor, platelet-derived endothelial cell growth factor, COX-2, PGE2, nitric oxide, NF-κB, MMP, and β-catenin [60,61,62].

One of the potential targets for n-3 LC-PUFAs in cancer suppression is angiogenesis, a process of new blood vessel formation within rapidly growing tumors. In 2008, Szymczak et al. showed that n-6 PUFAs stimulate, and n-3 LC-PUFAs inhibit major proangiogenic processes in human endothelial cells, including the induction of angiopoietin-2 (ANGPT2) and MMP-9, endothelial invasion, and tube formation, which are usually activated by the major n-6 PUFA, i.e., ARA. The COX-mediated conversion of PUFAs to prostanoid derivatives participated in the modulation of ANGPT-2 expression. These findings are consistent with the suggestion that PUFAs undergo biotransformation by COX-2 to lipid mediators that modulate tumor angiogenesis [63].

In order to well explain the molecular mechanisms of the involvement of PUFAs in tumoral angiogenesis, Hannafon et al. explored exosomes, an interesting cellular pathway, assuming that the intercommunication between cancer cells and their microenvironment is essential to tumor angiogenesis [63]. Exosomes are extracellular vesicles that are important mediators of intercellular communication, and play a role in promoting angiogenesis. Exosomes were collected from MCF7 and MDA-MB-231 breast cancer cells after treatment with DHA. They observed an increase in exosome secretion and exosome microRNA contents from the DHA-treated cells [63]. The expression of 83 microRNAs in the MCF7 exosomes was altered by DHA (>2-fold). The most abundant exosome microRNAs (let-7a, miR-23b, miR-27a/b, miR-21, let-7, and miR-320b) are known to have anti-cancer and/or anti-angiogenic activity. These microRNAs were also increased by DHA treatment in the exosomes from other breast cancer lines (MDA-MB-231, ZR751, and BT20), but not in exosomes from normal breast cells (MCF10A) [63]. When DHA-treated MCF7 cells were co-cultured with or their exosomes were directly applied to endothelial cell cultures, they observed an increase in the expression of these microRNAs in the endothelial cells. Furthermore, overexpression of miR-23b and miR-320b in endothelial cells decreased the expression of their pro-angiogenic target genes (PLAU, AMOTL1, NRP1, and ETS2), and significantly inhibited tube formation by endothelial cells, suggesting that the microRNAs transferred by exosomes mediate the anti-angiogenic action of DHA [63]. They concluded that DHA alters breast cancer exosome secretion and microRNA contents, which leads to the inhibition of angiogenesis. Their findings demonstrate that breast cancer exosome signaling can be targeted to inhibit tumor angiogenesis, and provide new insight into DHA’s anticancer action, further supporting its use in cancer therapy [63].

More recently, Aslan et al. showed a significant decrease in the expression of pro-angiogenic genes, including HIF1-α, TGF-β, SOX2, Snail1, Snail2, and VEGFR, in cells and also their secreted exosomes after treatment with DHA in normoxic and hypoxic conditions [64]. Moreover, the expression levels of tumor suppressor miRs, including miR-101, miR-199, and miR-342, were increased, and the expression levels of oncomiRs, including mir-382 and miR-21, were decreased after treatment with DHA in cells and exosomes [64]. They conclude that DHA can negatively affect the expression of pro-angiogenic genes and microRNA contents in breast cancer cells and their derived-exosomes in favor of the inhibition of angiogenesis. This interesting study demonstrated new insight into DHA’s anti-cancer activity to target not only breast cancer cells, but also their derived exosomes in order to stop tumor angiogenesis.

## 6. n-3 LC-PUFAs as Adjuvants of Chemotherapy and Protective Agents

Several studies have highlighted the potential of n-3 LC-PUFA as an adjuvant in the treatment of cancer, either as fish oil or as purified EPA/DHA (reviewed in: [65,66,67,68,69]). In breast cancer cell lines, DHA enhanced the cytotoxic effects of several anticancer agents through increased apoptosis, including doxorubicin [70,71,72], docetaxel [73], all-trans retinoic acid [74], and Trastuzamab [75]. 

In vivo experiments using a rat model of chemically-induced mammary tumorigenesis (i.e., N-methylnitrosurea-induced breast tumor) showed an enhanced reduction in tumor size by epirubicin (i.e., 40% greater reduction) when the diet contained approximately 0.7 g DHA per day [76]. Furthermore, this effect of DHA was related to a selective increase in oxidative damage, as evidenced by the elevated level of lipid hydroperoxide in the tumors [77]. In addition, in a similar model of a chemically-induced mammary tumor, DHA was shown to induce a microvascularization before and during docetaxel treatment, suggesting the potential usefulness of n-3 LC-PUFAs as an adjuvant strategy to enhance common anticancer drug efficiency [62]. The incorporation of DHA in tumor tissue during dietary DHA supplementation in rats was studied by Hajaji et al., and showed increased DHA-content in tumors from DHA-fed animals [78]. Interestingly, Newell et al. have shown that the combination of DHA with doxorubicin did not change the incorporation of DHA in either breast cancer cells (i.e., MCF-7 and MDA-MB-231 cell lines), lipid raft phospholipids, or even tumor phospholipids in nude mice implanted with MDA-MB-231 tumor in vivo [79]. This study also confirms the potential of DHA to enhance the anticancer effect of DOX by increasing the necrosis of tumors [79]. Besides, n-3 LC-PUFAs displayed interesting properties as sensitizing agents and multidrug-resistance revertants, as reviewed by Corsetto et al., 2017 [65]. These effects are related to n-3 LC-PUFA influence on membrane lipid composition, especially that of lipid raft, which leads to a significant modification of their physical-chemical properties altering the content and function of transmembrane proteins, such as receptors, growth factors, and ATP-binding cassette transporters [65]. 

Using a rat model of syngeneic xenograft, DHA was shown to enhance the antitumor activity of doxorubicin with increased oxidative stress in the tumor tissue [80], which is in accordance with the above results of Hajaji et al., 2012 [77]. Furthermore, this study highlighted that intra-tumoral doxorubicin concentration was increased with the DHA-supplemented diet without increasing the cardiotoxicity related to the drug [80]. This protective effect of n-3 LC-PUFAs against anthracycline drug cardiotoxicity has been observed in different cell and animal models of cancer, again supporting the value of n-3 LC-PUFAs associated with conventional breast cancer chemotherapy [81].

Finally, a phase II clinical trial showed that the addition of DHA to anthracycline-based therapy targeting metastatic breast cancer improved the chemotherapy outcome [49]. Furthermore, DHA has the potential to specifically sensitize tumor cells to chemotherapy or radiation therapy while protecting non-tumor cells [50,69]. Indeed, high incorporation of DHA during chemotherapy did not result in adverse side effects, and may improve the outcome of chemotherapy or radiotherapy [50,69].

## 7. Anti-Inflammatory Effect of n-3 LC-PUFAs and Cancer Prevention

**Figure 3:**
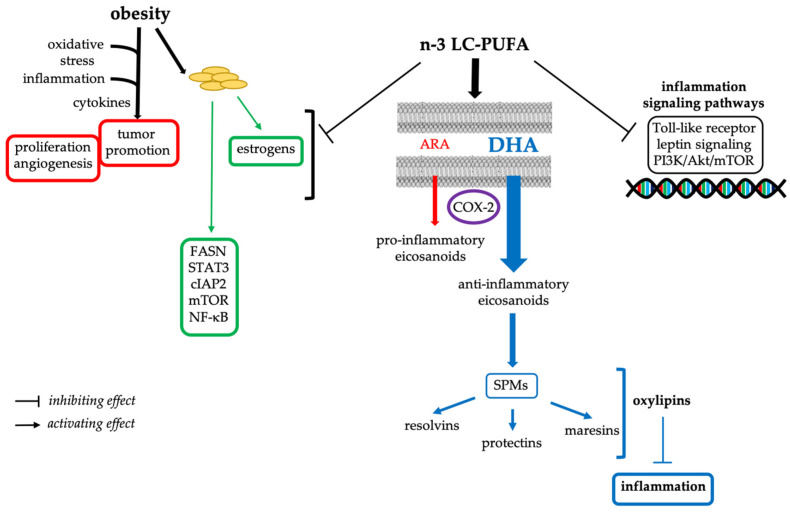
Anti-inflammatory effects of n-3 LC-PUFAs related to obesity and breast cancer.

The anti-inflammatory properties of n-3 LC-PUFAs have been known for a long time, and are thought to occur (i) by the replacement of ARA by DHA in cellular membranes, lowering the production of pro-inflammatory eicosanoids from ARA by COX and lipoxygenase; (ii) the fact that DHA is preferred to ARA as a substrate of COX-2 enzyme; and (iii) by the synthesis of lipidic specialized pro-resolving mediators (SPMs), namely, resolvins, protectins, and maresins, which are enzymatically oxygenated PUFAs (or oxylipins) acting as lipid mediators to suppress inflammation by clearing neutrophils and macrophages from inflammatory sites [82,83,84,85,86] (Figure 3). However, the synthesis of SPMs in humans is not clearly linked to n-3 LC-PUFA dietary supplementation [87]. As noted by Preethika et al. in an observational study of 102 breast cancer patients, high levels of ARA and low levels of n-3 LC-PUFAs resulted in a pro-inflammatory environment, which might contribute to breast cancer [88]. In addition, individuals with genetically determined lower fatty acid desaturase 1 (FADS1) activity due to the presence of the minor T allele will derive more benefit from n-3 LC-PUFAs, including a reduced risk of breast cancer, than those with higher FADS1 activity (G allele) [88].

The prevalence of obesity is increasing worldwide, and it is a critical risk factor for breast cancer, especially in post-menopausal women [89,90]. Indeed, women with obesity display highly proliferative and invasive tumors, resulting in a worse prognosis than lean women. Oxidative stress and systemic inflammation are known to be factors linking breast cancer to obesity through circulating cytokines and hormones that increase with weight gain [89]. The cytokines thus produced will stimulate the development of the tumor microenvironment, favorable to proliferation and angiogenesis. In addition to inflammatory cytokines, obesity increases estrogen production by adipocytes, which increases the risk of breast cancer. Thus, inflammation clearly appears to be the link between obesity and breast cancer, and the anti-inflammatory action of n-3 LC-PUFAs could reduce the risk of obesity-related breast cancer (Figure 3), not to mention the reduction of obesity itself through the consumption of n-3 LC-PUFAs [89,90]. The cross-talk between adipocytes and breast cancer cells was recently studied by Al-Jawadi et al. using conditioned media from 3T3-L1 adipocytes or human mesenchymal stem-cells-derived adipocytes and both MCF-7 and MDA-MB-231 breast cancer cell lines [91]. They confirmed the role of adipocytes in promoting breast cancer progression through increasing the expression of cancer-associated genes (i.e., FASN, STAT3, cIAP2, mTOR, NF-kB), which was canceled by the presence of EPA. The presence of EPA in the adipocyte culture also inhibited MCF-7 and MDA-MB-231 cell migration and, only for MCF-7 cells, glycolysis and glycolytic capacity [91].

The anti-inflammatory effect of dietary n-3 LC-PUFAs was also evidenced in human peripheral blood mononuclear cells collected from women with a high risk of breast cancer receiving dietary n-3 LC-PUFAs (four different doses) for 6 months of treatment [92]. This study showed that dietary supplementation with n-3 LC-PUFAs results in promoter hypermethylation in both genome-wide analysis and in candidate genes associated with inflammation-signaling pathways and breast cancer. Among the hypermethylated promoters, and thus transcriptionally inhibited, are those of the Toll-like receptor pathway, leptin signaling pathway, and focal adhesion/PI3K/Akt/mTOR pathway [92].

Finally, the anti-inflammatory effect of dietary n-3 LC-PUFAs may also protect against low-dose radiation, such as that used for mammography, and their effects on cancer cells and cancer progression [93].

## 8. Human and Clinical Studies Highlight the Benefit of n-3 LC-PUFAs Rich Diet

Until now, little is known on the real benefits of a n-3-LC-PUFAs-rich diet in breast cancer. Higher intakes of EPA and DHA from dietary sources were reported to be associated with a 25% reduction in breast cancer recurrence and improved overall mortality in a large cohort of over 3000 women with early stage breast cancer followed for a median of 7 years [94]. In addition, all-cause mortality was reduced among 1463 women newly diagnosed with first primary breast cancer with the higher quartile of dietary fatty fish and n-3 LC-PUFAs intake [95]. However, the effect of n-3 LC-PUFAs as dietary supplements before and/or during cancer treatment is somewhat controversial due to the lack of sufficiently robust randomized controlled clinical trials [96,97,98,99,100]. Besides, a very recent study concerning 250 registered n-3 LC-PUFA clinical trials and a screening for pertinence to cancer therapy showed a total of 171 entries [14]. Among these entries, ranging from 1995 to 2021, 24 concerned breast cancer trials using DHA + EPA/Rx showed that n-3 LC-PUFAs, with combinational treatment by adjuvant therapy or using single treatment in a preventive way or as treatment, (i) targeted SCD-1 for reducing breast cancer risk in obese postmenopausal women [101], (ii) changed n-3:n-6 ratio at the breast tissue in pre-menopausal women [102], and (iii) reduced incidence of paclitaxel-induced peripheral neuropathy [103]. In addition, the supplementation of newly diagnosed breast cancer patients with EPA and DHA led to a significant change in the composition of plasma fatty acids, and maintained the level of CD4+ T cells and serum levels of hsC-reactive protein, suggesting a beneficial effect on the immune system and a less active inflammatory response [104]. A healthy diet with n-3 LC-PUFAs significantly reduces serum IL-6 and TNFR-2 after body mass index adjustment in breast cancer patients, and might also act as anti-inflammatory compounds [103]. Clinical trials made with other cancer types, once an n-3 LC-PUFAs diet was performed, also showed a prominent and beneficial status [14]. Thus, in colorectal cancer, n-3 LC-PUFA preoperative treatment may provide a postoperative overall survival benefit [105], and an intravenous supply of n-3 LC-PUFA results in a rapid increase of EPA and DHA in plasma and of EPA in erythrocytes, and suggests a use to induce a rapid effect, especially in targeting inflammation [106]. The other effects that have been observed, regardless of the cancer types, were benefits against fatigue, cachexia, and reduction of infections, as well as the quality of life and the diminution of the toxicity on chemotherapy tolerability [14]. 

More recent studies have been carried out or are still recruiting volunteers to test numerous clinical effects hypotheses involving PUFAs, alone or in combination with other compounds. By querying the “ClinicalTrials.gov” database (https://clinicaltrials.gov/ accessed first on 21 February 2022 and last updated on 25 May 2022), which is a resource provided by the US National Library of Medicine containing privately and publicly funded international clinical studies, we found 680 clinical trials relating to PUFAs. Only 16 of the 680 studies are consistent with breast cancer, of which, 12 are less than 6 years old (at the completion date of the study), and only 2 trials have been completed and have results, whereas the other 10 trials are active or in the recruitment phase (Table 1).

Finally, fatty acid profiles in erythrocyte membranes appear as a relevant biomarker of breast cancer risk [108,109]. For example, this method was used to show that n-3 index was significantly lower in breast cancer patients than in healthy patients [110]. Furthermore, data from a multicenter lifestyle intervention study in women with hereditary breast cancer showed that the Mediterranean diet is associated with a likely favorable fatty acid composition of red blood cell membranes [111]. Buckland et al. have observed a significant and favorable decrease in the ratio of n-6 to n-3 LC-PUFAs after a short-term diet and exercise intervention in overweight/obese breast cancer survivors, suggesting positive dietary changes could be relevant for breast cancer prognosis [112]. In addition, the n-3 index was also related to lean mass and grip strength, suggesting that an n-3-LC-PUFA-enriched diet during and after cancer treatment may be causally linked with better muscle health and metabolic outcomes in breast cancer survivors [113]. Dietary intervention with n-3 LC-PUFAs may also have a beneficial impact on the fear of cancer recurrence and depressive symptoms among breast cancer survivors [114,115]. If fatty acid profiles were not significantly associated with breast cancer risk overall, some heterogeneity by body mass index was observed, and an inverse association of n-3 LC-PUFAs with breast cancer was observed among overweight/obese women [116].

## 9. Discussion and Perspectives

Taking the evidence discussed above, DHA is a safe, natural compound that can greatly improve the anticancer properties of anticancer drugs by additive or synergistic interactions [65,67,68]. In addition, n-3 LC-PUFAs reduced the risk of obesity-related breast cancer [90], and had protective effects toward the cardiotoxicity of anthracyclines, the most extensively used chemotherapeutics [81]. Thus, the current results of cohort studies and investigations in cell lines or animal models demonstrated that DHA could reduce tumor cell numbers by acting as soon as the cell begins its neoplastic transformation, through a decrease in proliferation and an increase in apoptosis [14,16,20]; this might explain the low level of breast cancer in populations with a high intake of DHA, such as Japanese people, Scandinavian people, people from Greenland, and people from Nunavut [12,50]. According to Stark et al. (2016), areas with high EPA + DHA blood levels (>6%) included the Sea of Japan, Scandinavia, Greenland, Nunavut, Mongolia, and some other areas with an indigenous population or populations not fully adapted to Westernized food habits [117]. More importantly, DHA may also reduce the invasive potential of certain cancers, which is one of the main complications with advanced cancers. It should be noted that some genetic determinant, i.e., single nucleotide polymorphism rs174537, may lead to an increased benefit of an n-3 LC-PUFA diet by lowering FADS1 [88]. In addition, and beyond breast cancer, it has been shown that n-3 LC-PUFAs may decrease cancer risk by affecting genetic variants of inflammatory pathways, oxidative stress, and tumor apoptosis [118]. This suggests the importance of (nutri-)genomics studies to identify genetic variants, and to increase the efficiency of n-3 LC-PUFA diet preventive action on breast cancer by a targeted or personalized therapeutic regimen to circumvent individual variation [88,118]. 

The source of n-3 LC-PUFAs may impact their efficiency, and plant-based n-3 LC-PUFA (mainly alpha-linolenic acid) appeared less potent in the prevention of mammary tumors than fish-oil-derived n-3 LC-PUFAs, which are mainly EPA and DHA [119]. However, fish oil is a limited resource due to overfishing and marine pollution, which has led to a decrease in fishery resources. The search for new sources rich in n-3 LC-PUFAs is, therefore, essential, and, thanks to their first place in the food chain and their low sensitivity to heavy metal contamination, microalgae could be an interesting alternative to fish oils. Microalgae rich in PUFAs have already shown their interest against cardiovascular pathologies, metabolic syndrome, and as anti-inflammatory, anti-obesity, and anti-cancer agents [16,19]. The liposome formulation of n-3 LC-PUFAs (DHA and EPA) may be a way to increase cellular uptake and protect n-3 LC-PUFAs from oxidation [120,121]. Such liposomal n-3 LC-PUFAs displayed anticancer effects like free DHA or EPA [120,121], but they also lead to the softening of cancer cells, which may improve the penetration and release of drugs into tumor cells, thus decreasing tumor resistance [121].

Besides, amine derivatives of n-3 LC-PUFAs, which are synthesized in vivo by enzymes such as phospholipases, hydrolysis, and, more specifically, by the amide hydrolase of fatty acids, are of increasing interest for their anticancer and anti-inflammatory effects [122]. These compounds, which include *N*-acyl-amines of DHA and EPA conjugated with ethanolamine, serotonin, alanine, serine, histidine, g-amino butyric acid, glutamic acid, or dopamine, could represent new signaling molecules [122]. For example, *N*-docosahexaenoyl ethanolamine (DHEA) and *N*-docosahexaenoyl serotonin (DHA-5-HT) have been shown to attenuate cytokine secretion by tumor-associated macrophages, which were obtained by the incubation of THP-1 monocytic cells with conditioned media of MCF-7 and MDA-MB-231 breast cancer cells [123]. This effect was reversed by an antagonist of peroxisome proliferator-activated receptor gamma (PPAR©), suggesting that PPARγ stimulation by n-3 LC-PUFAs or their endogenous derivative may lead to possible new strategies targeting both epithelial neoplastic cells and the tumor microenvironment [123].

Even when performed on the same cell line (notably the MDA-MB-231 line), the results obtained by the different authors with n-3 LC-PUFAs, and especially DHA, show a diversity of cellular mechanisms involved. However, the effects observed in the end are always similar, i.e., an anti-proliferative, pro-apoptotic, and anti-invasive effect, in almost all cell types and all types of cancers, even if the most studied remain colon cancer and breast cancer. These effects and the mechanisms observed have all been confirmed by animal studies. These observations reflect the heterogeneity of cancer cells, their metabolic and genetic disparity, and their instability. This diversity is ultimately reflected in the inter-individual variability of epidemiological studies. It is, therefore, this diversity that is to blame for the inconclusive nature of clinical studies with n-3 LC-PUFAs or dietary supplements rich in n-3 LC-PUFAs [96,97,98,99,100]. Another issue is the level of n-3 LC-PUFAs in animal and clinical studies, which has to be carefully determined to obtain clear anticancer effects [124]. Nevertheless, the results in cells and in vivo in animals all demonstrate the beneficial effect of n-3 LC-PUFAs and especially of an n-3/n-6 ratio favorable to n-3. As for the clinical studies, either they are clearly in favor of a beneficial effect of n-3 LC-PUFAs, or, at worst, they show no effect. In this case, it seems legitimate to proclaim that a diet rich in n-3 LC-PUFAs is preventive against breast cancer.

## 10. Conclusions

Even if the results in humans are not always very convincing due to numerous sources of inter-individual variations, n-3 PUFAs clearly appear to have many promising bioactivities, a real preventive effect against breast cancer, and a strong adjuvant potential to conventional therapies. 

## Figures and Tables

**Table 1:** List of clinical trials testing the effects of n-3 LC-PUFAS on breast cancer that are less than 6 years old.

NCT Number	Title	Status	Study Results	Phases	Enrollment	Study Type	Start Date
NCT05268913	Towards Early Detection of Breast Cancer in High-Risk Population	Not yet recruiting	No Results Available		120	Observational	1 March 2022
NCT03516253	Fish Oil and EPO in Breast Cancer	Active, not recruiting	No Results Available	Not Applicable	60	Interventional	20 February 2019
NCT04268134	Altering Lipids for Tolerance of Aromatase Inhibitor Therapy	Recruiting	No Results Available	Phase 2	75	Interventional	28 July 2020
NCT04716764	Dietary Advanced Glycation End Products, Inflammation, and Oxidative Stress in Breast Cancer Patients	Recruiting	No Results Available		32	Observational	3 March 2020
NCT01881048	Window of Opportunity Study Targeting the Inflammatory Milieu	Active, not recruiting	No Results Available	Early Phase 1	42	Interventional	8 December 2009
NCT01478477	Omega-3 Fatty Acids in Preventing Joint Symptoms in Patients With Stage I-III Breast Cancer Receiving Anastrozole, Exemestane, or Letrozole	Active, not recruiting	No Results Available	Not Applicable	44	Interventional	4 October 2011
NCT03949946	Is Lipid Mapping an Effective Early Detection Tool for Breast Cancer in High-risk Populations?	Recruiting	No Results Available		40	Observational	20 June 2019
NCT05048108	Remote Assessment of Cognition, Insulin Resistance, and Omega-3 Fatty Acid Biomarkers in Breast Cancer Survivors	Recruiting	No Results Available		80	Observational	18 January 2022
NCT02831582	Omega-3 Supplementation in Prevention of Aromatase Inhibitor-Induced Toxicity in Patients With Stage I-III Breast Cancer	Recruiting	No Results Available	Not Applicable	120	Interventional	12 October 2016
NCT03831178	Docosahexaenoic Acid (DHA) for Women With Breast Cancer in the Neoadjuvant Setting	Unknown status	No Results Available	Phase 2	52	Interventional	28 August 2019
NCT01869764	Omega-3 Fatty Acid in Treating Patients With Stage I-III Breast Cancer	Completed	Has Results(Not published)	Phase 2	57	Interventional	November 2013
NCT02352779	Omega-3 Fatty Acid in Reducing Cancer-Related Fatigue in Breast Cancer Survivors	Completed	Has ResultsPublished [107]	Not Applicable	108	Interventional	February 2015

Data accessed at https://clinicaltrials.gov/, on 21 February 2022 and last updated on 25 May 2022.

## Data Availability

Not applicable.
